# Daily home ECG monitoring for assessing the blanking period after catheter ablation in persistent atrial fibrillation

**DOI:** 10.1016/j.hroo.2025.08.006

**Published:** 2025-08-08

**Authors:** Daiki Shako, Keitaro Senoo, Arito Yukawa, Satoaki Matoba

**Affiliations:** 1 Department of Cardiovascular Medicine, Graduate School of Medical Science, Kyoto Prefectural University of Medicine, Kyoto, Japan; 2 Department of Cardiac Arrhythmia Research and Innovation, Graduate School of Medical Science, Kyoto Prefectural University of Medicine, Kyoto, Japan

**Keywords:** Atrial fibrillation, Electrocardiography, Early recurrence of atrial tachyarrhythmia, Late recurrence, Catheter ablation

**Key Findings**

▪ Early recurrence of atrial tachyarrhythmia (ERAT) occurring after day 64 postablation strongly predicts late recurrence (LR) of atrial fibrillation (AF).
▪ Frequent home electrocardiographic (ECG) monitoring during the blanking period (BP) improves recurrence detection. Patients performed a median of 3.36 ECG recordings per day using a home ECG device, enabling more granular analysis of ERAT timing compared with traditional ambulatory monitors.
▪ Shortening the BP from 90 to 64 days may be more appropriate. ERAT after day 64 was significantly associated with LR, challenging the conventional 90-day BP and suggesting a potential benefit of earlier intervention.
▪ Home ECG monitoring offers a feasible and noninvasive alternative to implantable loop recorders (ILRs). Despite not being a direct comparison study, findings are consistent with ILR studies and support the use of home ECG for long-term rhythm monitoring.
▪ Patient adherence is a critical factor in device-based AF monitoring. Of the 94 analyzed patients, 51 (54%) achieved *high adherence*, defined as recording ECGs on ≥80% of follow-up days, indicating feasibility with room for improvement.

Early recurrence after atrial fibrillation (AF) ablation is well known, and recent guidelines and statements have suggested that shortening the postablation blanking period (BP) from 12 to 8 weeks may be appropriate, as AF recurrence in the late stage of BP is strongly associated with late recurrence (LR).^1^

However, several studies using ambulatory electrocardiographic (ECG) monitors, such as Holter ECG and event recorders, have reported that the optimal threshold for early recurrence of atrial tachyarrhythmia (ERAT) in predicting LR of AF has been proposed at around 1 month.^2^ These monitoring methods are inherently limited by their recording duration and frequency.

In contrast, in a recently published study using an implantable loop recorder (ILR), early recurrences after 64 days represented the optimal timing threshold for predicting LRs of AF,^3^ showing a significant difference from the results of previous studies. This difference may be due to the variations in the recording frequency and timing of the ECG devices used in each study. Given this background, we hypothesized that frequent, multiple daily recordings using home ECG devices during the BP could improve monitoring performance and provide an ERAT threshold closer to that obtained in ILR studies.

**Figure 1:**
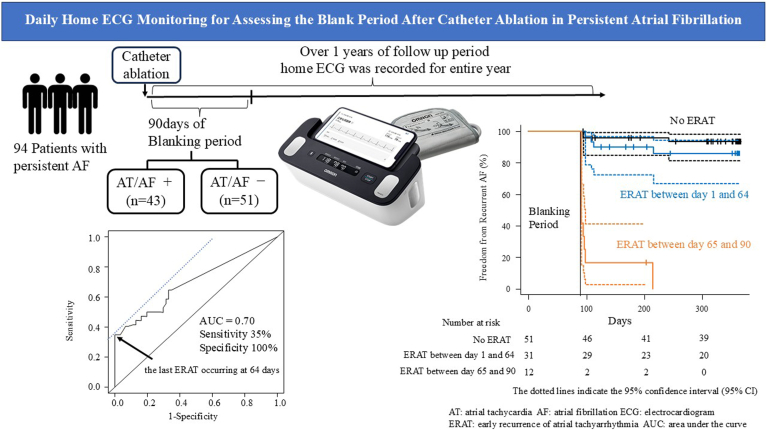
Summary of this study.

We performed our analysis using data from a multicenter observational study.^4^ To explain briefly, in this study, 94 patients with persistent AF undergoing ablation were enrolled. After undergoing ablation, they were instructed to measure their ECGs at home using the Complete device (ECG paired with a blood pressure monitor; Omron Healthcare). Recurrence of atrial tachyarrhythmia was defined as AF or atrial tachyarrhythmia documented by an ECG rhythm strip from Complete, a 12-lead ECG, or a 24-hour Holter ECG and had a duration of ≥30 seconds. Antiarrhythmic drugs were discontinued 3 months after ablation and were not resumed until recurrence was documented. Written informed consent was obtained from all individual participants included in the study. The research reported in this article adhered to the Helsinki Declaration guidelines. The study was approved by the Medical Ethics Review Committee of the Kyoto Prefectural University of Medicine (approval number: ERB-C-1347). Patients followed this protocol for a median of 78 days (interquartile range 57–87 years), resulting in a median of 240 ECGs (interquartile range 160–320 ECGs) or a median of 3.36 ECG recordings per day (interquartile range 2.87–3.82 ECG recordings per day). Over 1-year follow-up, 43 patients (45.7%) experienced ERAT and 33 patients (35.1%) had LR of AF. The risk of LR was higher in patients who experienced ERAT (hazard ratio 2.94; 95% confidence interval 1.42–6.08; *P* = .004). To determine the optimal timing for predicting LR of AF, receiver operating characteristic curve analysis identified day 64 as the optimal ERAT threshold for predicting LR, with an area under the curve of 0.70, a sensitivity of 0.35, and a specificity of 1.00. Patients with ERAT after day 64 had a significantly higher LR risk than did those with ERAT before day 64 or no ERAT (log-rank, *P* < .001) (Figure 1).

Frequent home ECG recordings revealed that ERAT occurring after 64 days was significantly associated with LR of AF, challenging the conventional 90-day BP. Shortening the BP from 3 to 2 months may improve the predictive accuracy for LR and allow earlier clinical interventions. Prior studies suggested differences in prognostic value between ERAT before 8 weeks and ERAT between 8 and 12 weeks, indicating the need for more precise time thresholds.

Traditional ambulatory ECG monitors, limited by continuous short-term recordings, often fail to detect intermittent arrhythmias and tend to define the BP at around 1 month. In contrast, home ECG devices combine the advantages of ambulatory monitors and ILRs. While not directly compared with ILRs, our findings align with ILR studies showing day 64 as an optimal ERAT predictor for LR. Home ECG devices offer noninvasive and cost-effective alternatives without the surgical burden of ILRs.

Several limitations of this study should be acknowledged. We did not collect data on symptom presence during ERAT or LR events, precluding correlation with subjective experiences. While our results support findings from ILR studies, we did not directly compare detection rates or outcomes in a head-to-head trial. Patient dropout and suboptimal device compliance are also important limitations. Of the 121 patients who underwent AF ablation, 94 were included in the final analysis after excluding patients because of hospital transfer (n = 1), withdrawal of consent (n = 15), investigator’s decision to discontinue follow-up (n = 3), and absence of any ECG measurements during the follow-up period (n = 8). *Adherence* was defined as achieving at least 1 ECG measurement per day on ≥80% of follow-up days using the Complete device. On the basis of this criterion, 51 patients were classified into the high-adherence group and 43 into the low-adherence group.^4^

In conclusion, our findings suggest that the standard 90-day cutoff for defining the BP may not be appropriate. Home ECG monitoring offers a noninvasive and cost-effective approach for long-term cardiac monitoring in postablation patients, providing valuable insights into AF recurrence patterns.
